# Achieving 3D Beamforming by Non-Synchronous Microphone Array Measurements

**DOI:** 10.3390/s20247308

**Published:** 2020-12-19

**Authors:** Liang Yu, Qixin Guo, Ning Chu, Rui Wang

**Affiliations:** 1 Institute of Vibration, Shock and Noise, State Key Laboratory of Mechanical System and Vibration, Shanghai Jiao Tong University, Shanghai 200240, China; liang.yu@sjtu.edu.cn; 2 College of Electronics and Information Engineering, Tongji University, Shanghai 201804, China; guo_qixin@tongji.edu.cn (Q.G.); ruiwang@tongji.edu.cn (R.W.); 3 College of Energy Engineering, Zhejiang University, Zheda Road 38, Hangzhou 310027, China

**Keywords:** 3D beamforming, non-synchronous measurements, sound sources localization

## Abstract

Beamforming technology is an essential method in acoustic imaging or reconstruction, which has been widely used in sound source localization and noise reduction. The beamforming algorithm can be described as all microphones in a plane simultaneously recording the source signal. The source position is then localized by maximizing the result of the beamformer. Evidence has shown that the accuracy of the sound source localization in a 2D plane can be improved by the non-synchronous measurements of moving the microphone array. In this paper, non-synchronous measurements are applied to 3D beamforming, in which the measurement array envelops the 3D sound source space to improve the resolution of the 3D space. The entire radiated object is covered better by a virtualized large or high-density microphone array, and the range of beamforming frequency is also expanded. The 3D imaging results are achieved in different ways: the conventional beamforming with a planar array, the non-synchronous measurements with orthogonal moving arrays, and the non-synchronous measurements with non-orthogonal moving arrays. The imaging results of the non-synchronous measurements are compared with the synchronous measurements and analyzed in detail. The number of microphones required for measurement is reduced compared with the synchronous measurement. The non-synchronous measurements with non-orthogonal moving arrays also have a good resolution in 3D source localization. The proposed approach is validated with a simulation and experiment.

## 1. Introduction

Sound source localization has high demand and exceptional value in applications such as automobiles [1], submarines [2], aircrafts [3,4], etc. Several methods have been proposed, such as beamforming and inverse methods [5,6], in which beamforming has evolved into one of the most important methods of sound source localization. The basic idea of beamforming is that by weighting each array element’s outputs, the expected maximum output power of the signal is directed to the position of the sound source. Different representations of the basis function also derive other classical methods, such as (1) near-field acoustical holography (NAH) [7,8,9]; (2) the inverse boundary element method (IBEM) [10,11,12]; (3) the equivalent source methods (ESM) [13,14,15]; (4) statistically optimal near-field acoustical holography (SONAH) [16,17]; and (5) the Helmholtz equation—least-squares method (HELS) [18]. The beamformer has a good resolution when the plane of the beamformer is parallel to the microphone plane. The spatial resolution of the planar beamformer deteriorated sharply when the plane is perpendicular to the microphone array. Most beamforming research is conducted on 2-dimentional (2D) because its model is typical and easy to explain the principle, which has been extensively studied in recent decades. Although most 2D beamforming algorithms can be directly extended to the 3-dimentional (3D) space domain for acoustic imaging [19,20,21], this simple extension method will cause problems, such as having a high computational complexity and difficulty in 2D parameter matching. On the other hand, 2D beamforming generally assumes that the distance between the source plane and the measurement plane is known. In contrast, 3D beamforming assumes that the source is distributed in a 3D space, and the distribution of the entire 3D sound source space is obtained by iterative scanning of the measured distance. In most studies, the implementation of 3D beamforming still uses planar array measurements, which leads to low spatial resolution problems. The 3D beamforming research is indispensable, achieving a high spatial resolution in both the lateral and normal directions of the planar array. The 3D model is more practical because the ability of great 3D sound source localization [22,23,24] is required in many industrial and national defense fields, especially in the aeroacoustics.

The research into 3D sound localization with a planar microphone array has been fully developed in recent years. A deconvolution approach of 3D DAMAS was developed to locate the distribution of gear noise sources, by Brooks et al. [20], which can improve the performance of the larger arrays at a higher frequency. A Fourier-based deconvolution with coordinate transformation and scanning technology were combined for 3D acoustic imaging by Xenaki et al. [22], which improves the normal resolution of the planar array but brings some side lobe pollutions. A compressive sensing algorithm was applied to 3D sound localization for obtaining a high-resolution source map by Ning et al. [25], which can provide more accurate results than conventional beamforming. The inverse solution strategies were exploited in the study of 3D acoustic mapping using a planar array, by Battista et al. [26], consisting of the equivalent source method and covariance matrix fitting method. Compared to the conventional beamforming approach, inverse strategies produce more accurate results. Sub-microphone arrays are added to the existing planar array to improve the 3D beamforming source map quality in some research. In 2013, four sub-microphone arrays, arranged around a wind tunnel, were used to detect aeroacoustic sources [27]. Padois and Berry [28] have compared the effects of various microphone array configurations on the 2D and 3D sound source localization. Two sub-arrays placed vertically were used to detect acoustic dipole sources in a 3D space [29]. These studies have found that adding a vertical sub-microphone array helps improve the planar array’s normal spatial resolution. There were 192 microphones in four arrays collecting sound signals at the same time in Padios et al.’s research [27]. The number of microphones in the combined array is larger when the microphone is dense in a single sub-array. Ping et al. proposed a 3D source localization model using a rigid spherical microphone array with spherical wave propagation [30], in which sparse Bayesian learning is used to perform the localization in 3D space. The above methods suffer from the problem of too many data acquisition channels or the high complexity of the algorithm. High-resolution 3D beamforming is still an important challenge and needs further study.

Non-synchronous measurements [31,32] can virtualize the microphone array into a large or high-density array by moving an array sequentially to record the signal at several locations. A sufficient number of fixed reference points are required to encode the phase relationships between the microphones in non-synchronous measurements. Antoni et al. [33] proposed a non-synchronous measurements method that does not require a reference point, in which the Bayesian probabilistic approach and the Expected-Maximization algorithm are used to reconstruct the source fields iteratively. The results of the non-synchronous measurements and the synchronous measurement have a similar accuracy. For this non-synchronous measurements method, Yu et al. [34] proposed fast iteration algorithms—including the augmented Lagrange multiplier (ALM) algorithm and alternative direction method of multipliers (ADMM) algorithm—to improve the iteration speed. These fast iteration algorithms can effectively recover the missing data in the cross-spectral matrix due to the non-synchronous measurements. The problem of non-synchronous measurements in 3D beamforming has received limited attention in the research literature, as most studies focused on 2D beamforming. Therefore, an interesting question arises: Is it possible to effectively improve the array’s spatial resolution by moving the planar microphone array in space?

**Figure 1:**
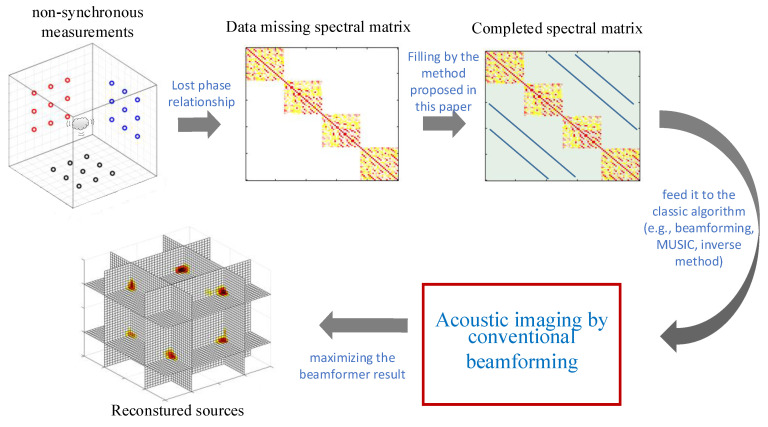
Algorithm flowchart of this paper. The data-missing spectral matrix is first obtained by non-synchronous measurements. Then the data-missing spectral matrix is filled with data to obtain the complete spectral matrix. Finally, acoustic imaging is achieved by conventional beamforming.

In this paper, the performance of 3D beamforming with the non-synchronous measurements is studied systematically. The prototype array configuration is optimized by moving a planar array in space when the sound sources are distributed in 3D space. Compared to conventional 3D beamforming, the number of microphones required for the measurement is reduced. The spherical basis is selected on the basis function instead of the Fourier basis when the beamforming expands to being 3D. The algorithm flowchart of this paper is shown in Figure 1. It is noted that conventional beamforming is just one convenient choice here and all the beamformers (i.e., MVDR and MUSIC [35,36]) that are based on the cross-spectral matrix can be applied in the proposed methods. The current paper is organized as follows. In Section 2, the non-synchronous measurements are developed in the context of 3D beamforming. The simulation results between a single synchronous measurement (i.e., one planar array) and non-synchronous measurements (i.e., a non-synchronous moving array) are compared in Section 3. In Section 4, the performance of the non-synchronous measurements and the synchronous measurement are discussed. In Section 5, an experiment is made to validate the performance of the non-synchronous measurements in 3D imaging. The main conclusions of this study are summarized in Section 6.

## 2. Forward Model of Acoustic Imaging and Acoustic Measurement

### 2.1. Conventional Beamforming

Denoting the sound pressure measured by the microphone at position r as p(r), p(r) is the sum of the particular pressure $p_{P}(r)$ and the random pressure $p_{N}(r)$. $p_{P}(r)$ is the sound pressure transmitted from the sound sources s at $r^{\prime }$ to the microphone at r, which can be obtained from the Green’s function $G(r,r^{\prime })$. $p_{N}(r)$ is the measurement noise, which is usually assumed to be a Gaussian distribution function [37,38]. For a given frequency ω, p(r) can be expressed as

(1) $$p(r,\omega )=p_{P}(r,\omega )+p_{N}(\omega )$$

where

(2) $$p_{P}(r,\omega )=\int_{r^{\prime }\in \Gamma }G(r,\omega |r^{\prime })s(r^{\prime },\omega )dr^{\prime },\;G(r,\omega |r^{\prime })=\frac{e^{jk{\left\|r-r^{\prime }\right\|}_{2}}}{4\pi {\left\|r-r^{\prime }\right\|}_{2}}$$

$G(r,\omega |r^{\prime })$ is the free-field Green’s function that describes the acoustic propagation between the sources at $r^{\prime }$ and the microphones at r. k=ω/c is the wavenumber that represents the number of radians per unit distance, and c is the wave velocity. The $\ell_{2}\text{-}\mathrm{norm}$ is defined as ${\left\|•\right\|}_{2}$.

Substituting Equation (2) into Equation (1), and re-writing the measured pressure signal in the form of the matrix, we can get

(3) $$p(r,\omega )=G(r,\omega |r^{\prime })s(r^{\prime },\omega )+p_{N}(\omega ).$$

Denoting the number of microphones as M and the number of the sources as S, the sizes are $p(r,\omega )\in {\mathbb{C}}^{M\times 1}$, $G(r,\omega |r^{\prime })\in {\mathbb{C}}^{M\times S},G=(g_{1},\ldots ,g_{s},\ldots ,g_{S})$, $s(r^{\prime },\omega )\in {\mathbb{C}}^{S\times 1}$, and $p_{N}(\omega )\in {\mathbb{C}}^{M\times 1}$. The cross-spectral matrix C(ω) can be obtained as

(4) $$C(\omega )=E\{p(r,\omega )p^{H}(r,\omega )\}=\frac{1}{I}\sum_{i=1}^{I}p_{i}(r,\omega )p_{i}^{H}(r,\omega )$$

where E{•} represent the mathematical expectation, ${\left(•\right)}^{H}$ represents the Hermitian transpose, I is the number of pressure snapshots, and the size is $C(\omega )\in {\mathbb{C}}^{M\times M}$.

Conventional beamforming is designed to locate sound sources by compensating for the time delay and amplitude attenuation between the microphones and the virtual source. The outcome of the beamformer $Q_{\mathrm{BF}}$ is calculated in terms of the cross-spectral matrix as

(5) $$Q_{\mathrm{BF}}=h_{n}^{H}C(\omega )h_{n}$$

The weights $h_{n}$ are designed to be independent of the array data or data statistics, which compensate for the time delays and amplitude attenuation of the forward propagation. It can be obtained as

(6) $$h_{n}=\frac{g_{n}}{{\left\|g_{n}\right\|}_{2}^{2}},n=1,\ldots ,S$$

The beamformer is designed to search for the source location by steering the microphone array. When the virtual source location coincides with the real source position, the outcome of the conventional beamforming gets the maximum. A more detailed description of the conventional beamforming can be found in Chu’s work [35].

### 2.2. Non-Synchronous Measurements Theory

**Figure 2:**
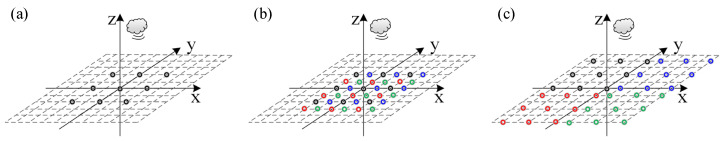
Schematic diagram of the non-synchronous measurements in a 2D space: (**a**) measurement with a planar microphone array; (**b**) non-synchronous measurements’ microphone array for a denser array; (**c**) non-synchronous measurements’ microphone array for a bigger array. The circle “○” represents a microphone, and the same color represents the microphones in the same test.

Unlike synchronous measurement, in which all microphones acquire data simultaneously, non-synchronous measurements receive data by moving the microphone array under the assumption of a stable sound field. There is evidence that non-synchronous measurements are effective in 2D beamforming. For non-synchronous measurements, a denser or larger virtual array can be obtained by moving the microphone array in a 2D space, as shown in Figure 2. In denser virtual arrays, the working frequency of the beamforming algorithms is expanded; in larger virtual arrays, the entire radiating object is also better covered [39].

**Figure 3:**
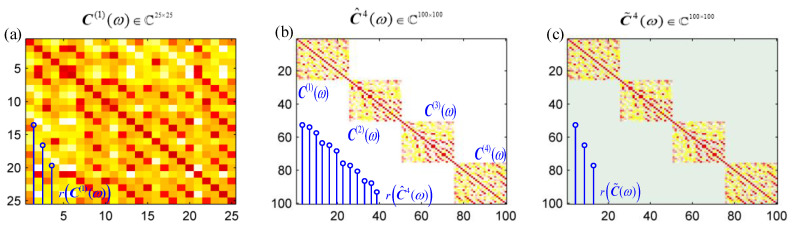
(**a**) $C^{\left(1\right)}(\omega )\in {\mathbb{C}}^{25\times 25}$ in one single measurement; $r(C^{\left(1\right)})$ is the rank of $C^{\left(1\right)}(\omega )$. (**b**) ${\hat{C}}^{4}(\omega )\in {\mathbb{C}}^{100\times 100}$ by arranging $C^{\left(i\right)}(\omega ),i=1,2,3,4$ in the diagonal block position; $r({\hat{C}}^{4})$ is the rank of ${\hat{C}}^{4}(\omega )$. (**c**) ${\tilde{C}}^{4}(\omega )\in {\mathbb{C}}^{100\times 100}$ with the non-synchronous measurements by implementing the missing data at the non-diagonal block; $r({\tilde{C}}^{4})$ is the rank of ${\tilde{C}}^{4}(\omega )$.

The sound source localization is based on the beamforming method, whether the synchronous or non-synchronous measurements. The cross-spectral matrix C(ω) is the main difference between the synchronous and non-synchronous measurements. For synchronous measurements, C(ω) is a M×M full matrix. The rank of C(ω) is equal to the number of uncorrelated sources S. For the non-synchronous measurements, a complete cross-spectral matrix is obtained, no longer due to the moving microphone array. The obtained matrix ${\hat{C}}^{P}(\omega )$ will lose the non-diagonal block elements’ information when the cross-spectral matrix obtained from each measurement $C^{\left(i\right)}(\omega )$ is placed on the diagonal block of a larger matrix. The superscript P refers to the total number of the non-synchronous measurements and the superscript i refers to the i-th measurement. ${\hat{C}}^{P}(\omega )$ is a MP×MP matrix. When the elements at the non-diagonal position of ${\hat{C}}^{P}(\omega )$ are set to zero in the absence of references, the rank of ${\hat{C}}^{P}(\omega )$, $r({\hat{C}}^{P}(\omega ))$, is equal to the product of the number of the sources and the number of moves, SP. The rank of the cross-spectral matrix is considered to be the number of sources. The non-synchronous measurements method is located in the source position by supplementing the missing data in ${\hat{C}}^{P}(\omega )$. The accomplished cross-spectral matrix ${\tilde{C}}^{P}(\omega )$ has the same rank as $C^{\left(i\right)}(\omega )$ in one measurement. There is an example to clarify the spectral matrix issues in the non-synchronous measurements: assuming that the microphone array includes 25 microphones and the scanning plane contains three sound sources. Figure 3a shows the cross-spectral matrix $C^{\left(1\right)}(\omega )$ in one measurement. It is a 25×25 full matrix, and the rank of $C^{\left(i\right)}(\omega )$, $r(C^{\left(i\right)})$, is equal to 3. If the prototype array moves four times, ${\hat{C}}^{4}(\omega )$ (as shown in Figure 3b) is obtained by rearranging the $C^{\left(i\right)}(\omega ),i=1,2,3,4$ of the single measurement in the diagonal block position. ${\hat{C}}^{4}(\omega )$ is a (25×4)×(25×4) matrix, with data missing at the non-diagonal positions. The rank of ${\hat{C}}^{4}(\omega )$, $r({\hat{C}}^{4})$, is equal to 12 (i.e., 3×4). Figure 3c shows the target spectral matrix, ${\tilde{C}}^{4}(\omega )$. The missing data were supplemented by the non-synchronous measurements and the rank of ${\tilde{C}}^{4}(\omega )$, $r({\tilde{C}}^{4})$, was restored to 3.

The core problem in the non-synchronous measurements is the data-missing spectral matrix completion problem. The process of finding a full cross-spectral matrix can be described as

(7) $$\begin{array}{l} \mathrm{find}\;\tilde{C}\in {\mathbb{C}}^{MP\times MP}\;such\;that\{\begin{array}{l} r(\tilde{C})=S\\ {\left\|A(\tilde{C})-\hat{C}\right\|}_{F}\leq \varepsilon_{1}\\ \|\Psi \tilde{C}\Psi^{H}-\tilde{C}{\|}_{F}\leq \varepsilon_{2}\\ {\tilde{C}}^{H}=\tilde{C}\geq 0 \end{array} \end{array}$$

The four additional constraints are explained as follows:

1. The 1st constraint: $r(\tilde{C})=S$ ensures that the rank of the reconstructed cross-spectral matrix is still equal to the number of the sources S.
2. The 2nd constraint: A(•) denotes the sampling operator that gets the elements in the diagonal block of a matrix, and the size is $A(\tilde{C})\in {\mathbb{C}}^{MP\times MP}$, which is identical to $\tilde{C}\in {\mathbb{C}}^{MP\times MP}$. $||A(\tilde{C})-\hat{C}|{|}_{F}\leq \varepsilon_{1}$ ensures that the difference between $A(\tilde{C})$ and $\hat{C}$ in the Frobenius norm is less than a given tolerance $\varepsilon_{1}$.
3. The 3rd constraint: Ψ is a projection matrix. $\Psi \tilde{C}\Psi^{H}=E\{\Psi \tilde{P}{\tilde{P}}^{H}\Psi^{H}\}$ is the cross-spectral matrix of $\Psi \tilde{P}$, where the projected matrix $\Psi \tilde{P}$ can be the smooth pressure [40] of the non-synchronous measured pressure $\tilde{P}$. To ensure the acoustic field’s spatial continuity, $||\Psi \tilde{C}\Psi^{H}-\tilde{C}|{|}_{F}=||E\{\Psi \tilde{P}{\tilde{P}}^{H}\Psi^{H}\}-E\{\tilde{P}{\tilde{P}}^{H}\}|{|}_{F}\leq \varepsilon_{2}$ is added here, and the difference in the cross-spectral matrix between $\Psi \tilde{P}$ and $\tilde{P}$ is required to be smaller than a given tolerance $\varepsilon_{2}$. The detailed discussion on this constraint is addressed in Section 2.2.
4. The 4th constraint: ${\tilde{C}}^{H}=\tilde{C}\geq 0$ ensures that both ${\tilde{C}}^{H}$ and $\tilde{C}$ are positive semi-definite matrixes.

It can be seen from the 1st constraint, the eigenvalue vector of the cross-spectral matrix should be S-sparse. The number of non-zero eigenvalues is S, equal to the number of sound sources. The rank estimation of a matrix is still a difficult problem. An alternative model has been proposed to solve this problem: the cross-spectral matrix is considered to be a full rank matrix, with a few dominant eigenvalues. This method considers the eigenvalue spectrum to be “weak sparse”, in which the sorted eigenvalue elements of the spectral matrix decay rapidly according to the power law, rather than “complete S-sparse”. Equation (7) can be realized in another form of constrained optimization as

(8) $$\begin{array}{ll} \underset{\tilde{C}}{\mathrm{minimize}\;} & ||\tilde{C}|{|}_{*}\\ \mathrm{subject}\;\mathrm{to} & ||A(\tilde{C})-{\hat{C}}^{m}|{|}_{F}\leq \varepsilon_{1}\\ & ||\Psi \tilde{C}\Psi^{H}-\tilde{C}|{|}_{F}\leq \varepsilon_{2}\\ & {\tilde{C}}^{H}=\tilde{C}\geq 0 \end{array}$$

where $||•|{|}_{*}$ denotes the nuclear norm of a matrix, which is defined as the sum of the eigenvalues $\lambda_{i}$, so $||•|{|}_{*}=\sum_{i=1}^{i=MP}\lambda_{i}(•)$. The objective function $\underset{\tilde{C}}{\mathrm{minimize}}\;||\tilde{C}|{|}_{*}$ is to seek $\tilde{C}$ with the minimum nuclear norm.

**Figure 4:**
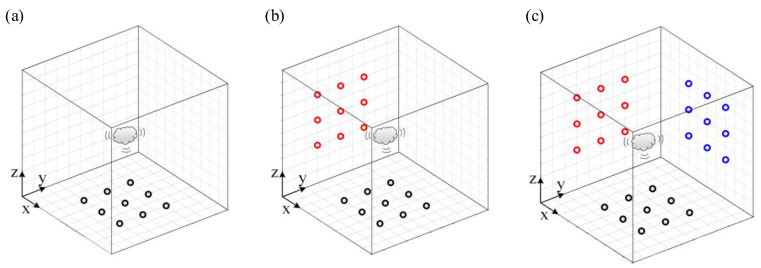
(**a**) Non-synchronous original microphone array in the x–y plane (black circles); (**b**) non-synchronous moving microphone array in space from the x–y plane to the y–z plane (red circles); (**c**) non-synchronous moving microphone array in space from the x–y plane to the x–z plane (blue circles). The circle “○” represents a microphone, and the same color represents the microphones in the same test.

2D beamforming generally assumes that the distance between the source plane and the measurement plane is known, and the non-synchronous measurements move the microphone array on one plane, making the virtual microphone array denser or larger (seen in Figure 2). 3D beamforming assumes that the source is distributed in a 3D space, and the distribution of the entire 3D sound source space is obtained by iterative scanning of the measured distance. In most studies, the implementation of 3D beamforming still uses planar array measurements, which leads to low spatial resolution problems. In this paper, the non-synchronous measurements are used to measure 3D space, in which the measurement array envelops the 3D sound source space to improve the resolution of the 3D space. As shown in Figure 4, the original array is located in the x–y plane, and then the array can be moved sequentially to the y–z plane and x–z plane, such that the scattering object is surrounded by the virtual microphone array in three planes. This study aims to extend the application of non-synchronous measurements in 3D beamforming.

### 2.3. Spatial Basis and Spatial Continuity of the Acoustic Field

**Figure 5:**
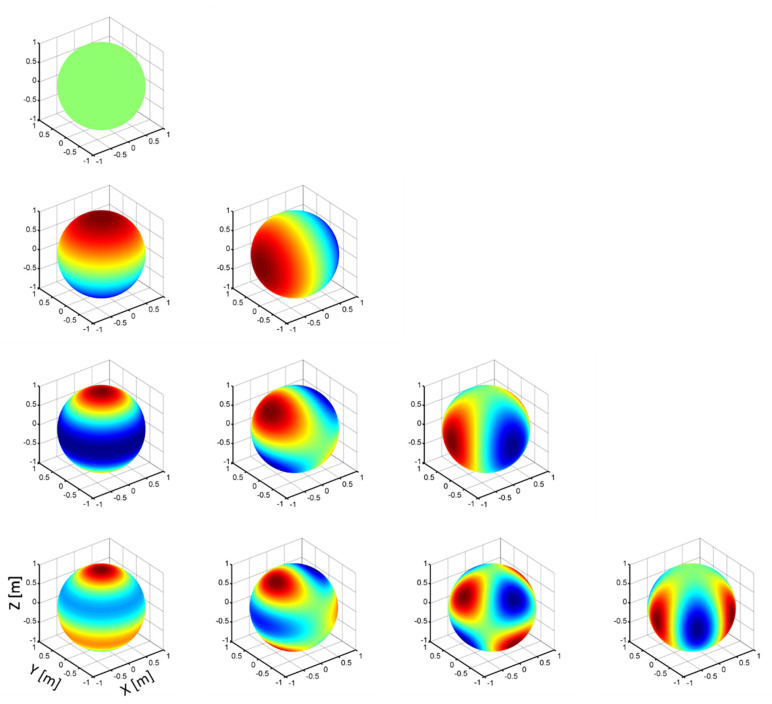
Spherical harmonics. The real spherical harmonics $Y_{l}^{m}(\theta ,\varphi )$ for l=0,1,2,3 (top to bottom) and m=0,1⋯l (left to right). The value of the spherical harmonic function is represented by color, in which the maximum value is characterized by red, and the minimum value is represented by blue.

The sound pressure signal captured by the microphone P can be expressed using a set of spatial bases as

(9) $$P=\sum_{i=1}^{n}\phi_{i}(r)\vartheta_{i}=\Phi \vartheta$$

where Φ is the spatial basis vector and ϑ is the coefficients vector. $\phi_{i}(r)$ and $\vartheta_{i}$ are the i-th basis and the corresponding coefficient, respectively. The Fourier basis $\Phi (x,y)=e^{i(k_{x}x+k_{y}y)}$ is chosen for 2D beamforming, where x and y are the microphones’ coordinates, and $k_{x}$ and $k_{y}$ are the wavenumbers along the x and y directions. For 3D beamforming, a 3D spatial basis should be considered. In this paper, spherical harmonics are chosen (as shown in Figure 5), and its complete orthonormal form in polar coordinates (r,θ,φ), $Y_{l}^{m}(\theta ,\varphi )$ of order l and degree m, can be written as

(10) $$Y_{l}^{m}(\theta ,\varphi )={\left(i\right)}^{m+|m|}\sqrt{\frac{\left(2l+1\right)}{4\pi }\frac{(l-|m|)!}{(l+|m|)!}}P_{l}^{m}(\cos\theta )e^{im\varphi }$$

where $l\in R^{+}$, |m|≤l. $P_{l}^{m}(•)$ is the associated Legendre function:

(11) $$P_{l}^{m}(x)={\left(1-x^{2}\right)}^{|m|/2}\frac{d^{\left|m\right|}}{dx^{\left|m\right|}}P_{l}(x)$$

$P_{l}(•)$ is the Legendre polynomial of degree l:

(12) $$P_{l}(x)=\frac{1}{2^{l}l!}\frac{d^{l}}{dx^{l}}{\left(x^{2}-1\right)}^{l}$$

The negative order spherical harmonics $Y_{l}^{-m}(\theta ,\varphi )$ can be obtained by rotating $90^{o}/m$ around the z-axis relative to the positive harmonics. The pressure signals at one microphone P(r,θ,φ) can be re-written in forms of spherical harmonics as

(13) $$P(r,\theta ,\varphi )=\sum_{l=0}^{n}\sum_{m=-l}^{l}\theta_{l}^{m}(r)Y_{l}^{m}(\theta ,\varphi )$$

where $\theta_{l}^{m}(r)$ is the coefficient.

Assuming the acoustic field’s spatial continuity, the reconstructed acoustic field should be smooth, and the sound pressures measured by the two adjacent microphones should have comparable levels. Such constraints should be included in the optimization model. Since the best approximation of vector P in subspace R(Φ) is its projection vector ${\mathrm{Proj}}_{R(\Phi )}P$, we need to get the orthogonal projection matrix on the subspace R(Φ). According to Equation (9), the coefficient ϑ can be obtained from the measured pressure P as

(14) $$\vartheta =\Phi^{†}P$$

where † denotes the pseudo-inverse of a matrix based on the fact that Φ is not generally invertible. The smoothed pressure $\tilde{P}$ can be obtained by

(15) $$\tilde{P}=\Phi \Phi^{†}P=\Psi P,$$

where $\Psi =\Phi \Phi^{†}$ is the orthogonal projection matrix, and $\tilde{P}={\mathrm{Proj}}_{R(\Phi )}P=\Phi \Phi^{†}P$ is the projection of P on space R(Φ). The cross-spectral matrix of the smoothed pressure $\tilde{C}$ is given by

(16) $$\tilde{C}=E(\tilde{P}{\tilde{P}}^{H})=E(\Psi PP^{H}\Psi^{H})=\Psi E(PP^{H})\Psi^{H}=\Psi C\Psi^{H}$$

If the smoothed pressure $\tilde{P}$ is already in the space R(Φ), the projection of $\tilde{P}$ in space R(Φ) should be itself. The constraint $||\Psi \tilde{C}\Psi^{H}-\tilde{C}|{|}_{F}\leq \varepsilon_{2}$ is included in the optimization model, where $\Psi \tilde{C}\Psi^{H}$ denotes the re-projection result and $\tilde{C}$ denotes the smoothed result. $\Psi =\Phi \Phi^{†}$ has encoded the microphone position information into the matrix $\tilde{C}$.

## 3. Simulation Results

For conventional 2D beamforming, the plane where the sound sources are located is discretized into N grids with known positions. For 3D acoustic imaging, the observation zone will be extended from one plane to a rectangular box. In the current numerical experiments, the rectangular box’s center to be scanned is at the origin, and the size is 0.4 m×0.4 m×0.4 m. The scanned box is discretized uniformly by a 41×41×41 grid with a distance of 0.01 m. The six-point sources with equivalent unit magnitudes are located at (−0.1, −0.1, −0.1), (−0.1, 0.1, −0.1), (0.1, −0.1, −0.1), (−0.1, 0.1, 0.1), (0.1, 0.1, 0.1), and (0.1, −0.1, 0.1) (m). These sound sources are located at the vertex of an imaginary cube with the center at the origin and the side length of 0.2 m. The signal of the source is generated by random white noise. The prototype planar array with 56 microphones distributed by the Archimedes spiral is initially located in the z = −0.5 m plane, and the center of the planar array is located at (0, 0, −0.5) (m). The frequency of beamforming is set to 4000 Hz, and the signal-to-noise ratio (SNR) is set to 20 dB. To illustrate the advantages of the 3D beamforming with non-synchronous measurements, the results of 3D imaging by the conventional beamforming and the non-synchronous measurements are compared in the following sections. The results of 3D imaging by the conventional beamforming with the original planar array are given in Section 3.1. As a comparison, the results of the 3D imaging by the non-synchronous measurements with orthogonal and non-orthogonal moving arrays are shown, respectively, in Section 3.2 and Section 3.3. The orthogonal moving arrays mean that the initial microphone array and the moving microphone array are perpendicular to each other. The non-orthogonal moving arrays mean a certain angle between the microphone array before and after moving.

### 3.1. 3D Imaging by the Conventional Beamforming with a Planar Array

**Figure 6:**
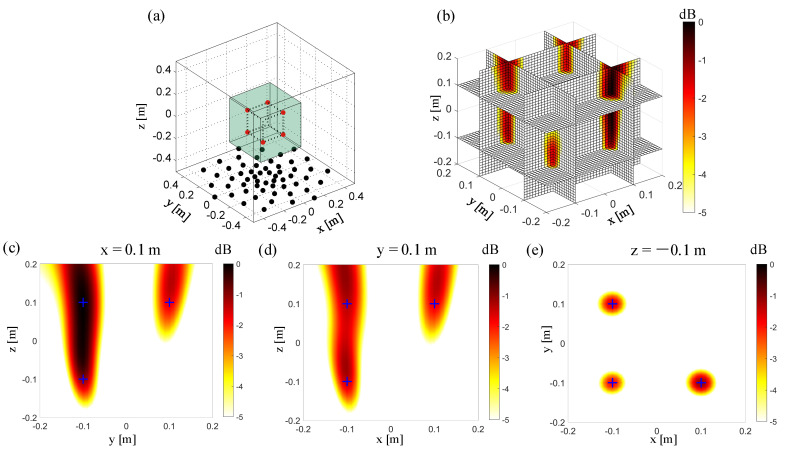
3D Beamforming result with a single planar array. (**a**) The simulation setup. The red points “**·**” represent the sound sources. The other same color represents the microphones in the same test. The black points “**·**” represent the microphones in a single planar array test. (**b**) 3D beamforming map. (**c**) The beamforming map at the x = 0.1 m slice. (**d**) The beamforming map at the y = 0.1 m slice. (**e**) The beamforming map at the z = −0.1 m slice.

Figure 6a shows the microphone array’s geometry and the location of the sound sources in the numerical simulation. The microphone array is set to be z = −0.5 m, and the center of the array is set to (x = 0 m, y = 0 m, z = −0.5 m). The scanned box is shown as partially transparent to allow visualization of the sound sources. Figure 6b shows the source of power maps in the observation zone, which has been normalized by the maximum value. Six slices are presented on this figure to get a better view of the sound sources location. The simulated sound sources are located at the intersection of these slices. Figure 6c–e respectively show the sources power maps at three typical slices (x = 0.1 m, y = 0.1 m, z = −0.1 m). The positions of the simulated sound sources are marked with “+” on the figures. Figure 6e shows, on the z = −0.1 m slice, that, parallel to the microphone array, conventional beamforming locates the sound sources well, and has a good spatial resolution in both the x and y directions. As shown in Figure 6c,d, on x = 0.1 m and y = 0.1 m, for the slices perpendicular to the microphone array, it is difficult to distinguish the sound source’s location by conventional beamforming. The main lobes of these sound sources are merged into the perpendicular directions, which leads to poor spatial resolution so that the sound source’s position cannot be accurately located.

### 3.2. 3D Imaging by the Non-Synchronous Measurements with Orthogonal Moving Arrays

**Figure 7:**
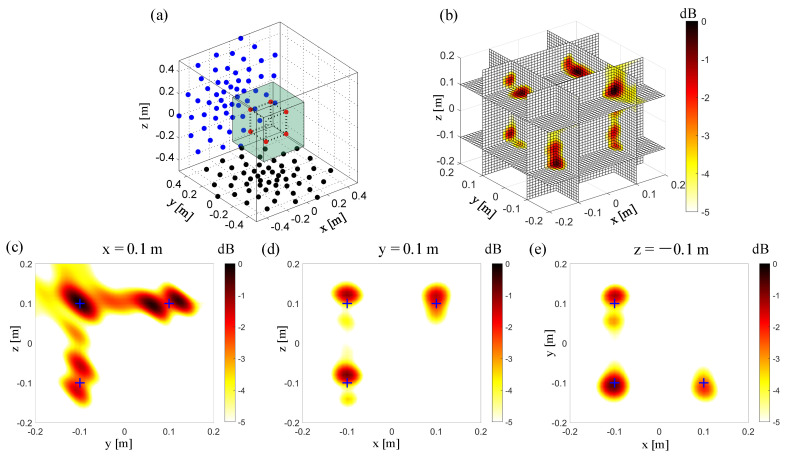
3D Beamforming results from the non-synchronous measurements, orthogonally moving the array once. (**a**) The simulation setup. The red points “**·**” represent the sound sources. The other same color represents the microphones in the same test. The black points “**·**” represent the microphones in a single planar array test. The blue points “**·**” represent the microphones in the test with the orthogonal moving array, moved here for the first time. (**b**) 3D beamforming map. (**c**) The beamforming map at the x = 0.1 m slice. (**d**) The beamforming map at the y = 0.1 m slice. (**e**) The beamforming map at the z = −0.1 m slice.

The orthogonal moving arrays mean that the moving microphone array is perpendicular to the plane of the previously tested microphone. Figure 7a shows the microphone array configuration when the prototype array is moved once. The initial microphone array is located on the z = −0.5 m plane, which is consistent with the array position in Section 3.1, and the moving microphone array is located on the y = −0.5 m plane. The geometric center of the moving microphone array is located on (x = 0 m, y = −0.5 m, z = 0 m). The results in the 3D zone and typical slices are shown in Figure 7b–e. Comparing the results in Figure 6d and Figure 7d, the orthogonally moving the microphone array one time can improve the resolution in the z-direction of the x-z plane of the initial prototype array. The positions of the sound sources in the Z direction are not distinguished well due to the merging of the main lobes in Figure 6d, while the two sound source positions in the Z direction are located accurately in Figure 7d. By comparing the results in Figure 6c and Figure 7c, the y–z plane’s resolution is slightly improved when the microphone array is moved orthogonally once, the resolution in the Z direction is not enough to locate the positions of the sound sources.

**Figure 8:**
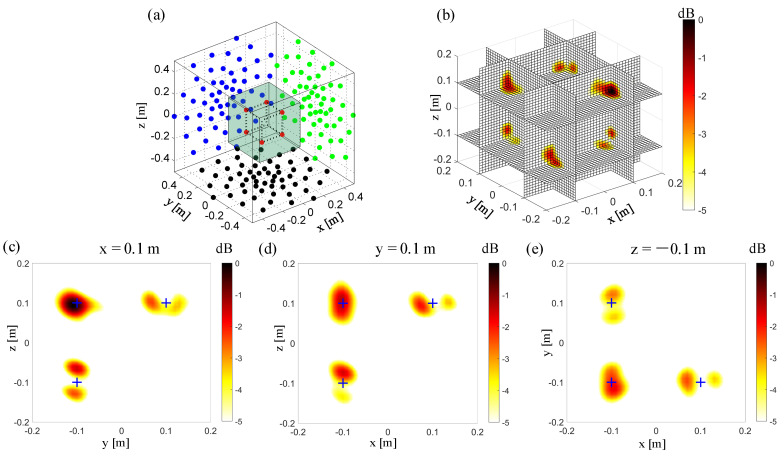
3D Beamforming results from the non-synchronous measurements, orthogonally moving the array twice. (**a**) The simulation setup. The red points “**·**” represent the sound source. The other same color represents the microphones in the same test. The black points “**·**” represent the microphones in a single planar array test. The blue points “**·**” represent the microphones in the test with the orthogonal moving array, moved here for the first time. The green points “**·**” represent the microphones in the test with the orthogonal moving array, moved for the second time. (**b**) 3D beamforming map. (**c**) The beamforming map at the x = 0.1 m slice. (**d**) The beamforming map at the y = 0.1 m slice. (**e**) The beamforming map at the z = −0.1 m slice.

In this simulation case, the microphone array moves orthogonally twice. It moves vertically again based on the simulation case in Figure 7. In Figure 8a, the “third” microphone array is located on the x = 0.5 m plane, and the microphone array’s center is located at (x = 0.5 m, y = 0 m, z = 0 m). The non-synchronous measurements of using three orthogonal moving microphone arrays can achieve good spatial resolution in the x, y, and z directions. Figure 6, Figure 7 and Figure 8 show that the non-synchronized measurement by orthogonally moving the microphone array can overcome the lack of spatial resolution in the normal direction of the planar array. The planar microphone array can only guarantee the spatial resolution on the plane parallel to the microphone array. The microphone array is moved orthogonally and continuously twice, where a high spatial resolution can be obtained in all directions during 3D acoustic imaging.

### 3.3. 3D Imaging by the Non-Synchronous Measurements with Non-Orthogonal Moving Arrays

**Figure 9:**
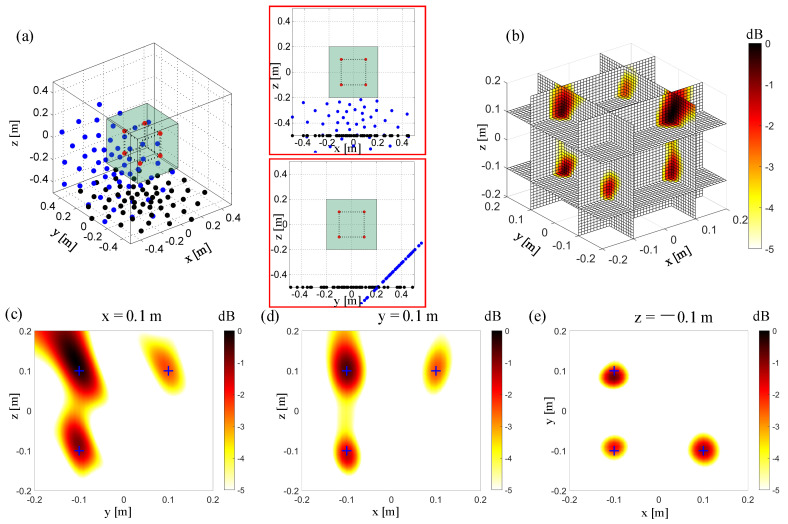
3D Beamforming results from the non-synchronous measurements, non-orthogonally moving the array once. (**a**) The simulation setup. The red points “**·**” represent the sound sources. The other same color represents the microphones in the same test. The black points “**·**” represent the microphones in a single planar array test. The blue points “**·**” represent the microphones in the test with the non-orthogonal moving array, moved here for the first time. The first subfigure represents the view in the x–z plane, and the second subfigure represents the view in the y–z plane. (**b**) 3D beamforming map. (**c**) The beamforming map at the x = 0.1 m slice. (**d**) The beamforming map at the y = 0.1 m slice. (**e**) The beamforming map at the z = −0.1 m slice.

In the previous simulation configuration, the microphone array is always moving orthogonally. Due to limitations in measurement space or movement error, it is not always guaranteed that the microphone array remains orthogonal. Figure 9a shows the microphone array configuration when the prototype array is rotated 45° along the *x*-axis. The miniatures in the upper-right and lower-right corners are the x–z and y–z views of the microphone array configuration, respectively. The initial microphone array is located at the z = −0.5 m plane. The “second” microphone array is at an angle of 45° to the z = −0.5 m plane, and its center is 0.5 m away from the origin point (0, 0, 0). Figure 9 shows that the “second” microphone array still improves the resolution in the Z direction in the y = 0.1 m plane. Compared to the results of the orthogonally moving microphone array in Figure 7, the main lobes of the sources at y = 0.1 is relatively larger. As shown in Figure 9c, the extension direction of the main lobes in the x = 0.1 plane changes a bit. The resolution in the x = 0.1 m plane needs to be improved by further moving the microphone array.

**Figure 10:**
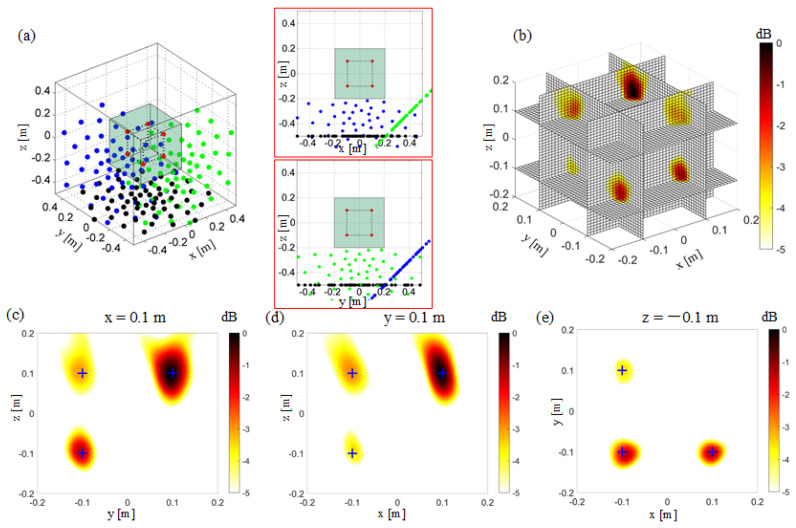
3D Beamforming results from the non-synchronous measurements, non-orthogonally moving the array twice. (**a**) The simulation setup. The red points “**·**” represent the sound sources. The other same color represents the microphones in the same test. The black points “**·**” represent the microphones in a single planar array test. The blue points “**·**” represent the microphone in the test with the non-orthogonal moving array, moved here for the first time. The green points “**·**” represent the microphone in the test with the non-orthogonal moving array, moved for the second time. The first subfigure represents the view in the x–z plane, and the second subfigure represents the view in the y–z plane. (**b**) 3D beamforming map. (**c**) The beamforming map at the x = 0.1 m slice. (**d**) The beamforming map at the y = 0.1 m slice. (**e**) The beamforming map at the z = −0.1 m slice.

The microphone array is moved again based on the previous analysis. The configuration of the microphone array after moving is shown in Figure 10a. The miniatures in the upper-right and lower-right corners are the x–z and y–z views of the microphone array configuration. The “third” microphone array rotates the initial microphone array by −45° on the *y*-axis. Its center still keeps 0.5 m away from the origin point (0, 0, 0). The results show that the resolution in the x = 0.1 m plane is improved. The main lobes of sound sources can be distinguished in the Z direction, and the sound sources localization effect is relatively better than that in Figure 6 and Figure 9. We can conclude that the non-synchronous measurements in the 3D space can improve the spatial resolution. Compared with Figure 8e, Figure 10e shows the more accurate result of the sound source localization because in the x–y slice the density of the microphone array increases.

## 4. Comparison between the Synchronous Measurements and Non-Synchronous Measurements

**Figure 11:**
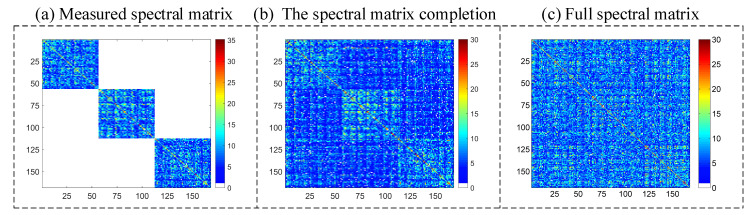
Comparison of the spectral matrix between the synchronous and non-synchronous measurements. (**a**) Measured cross-spectral matrix. (**b**) The spectral matrix completion from the non-synchronous measurements. (**c**) Full spectral matrix from the synchronous measurements.

**Figure 12:**
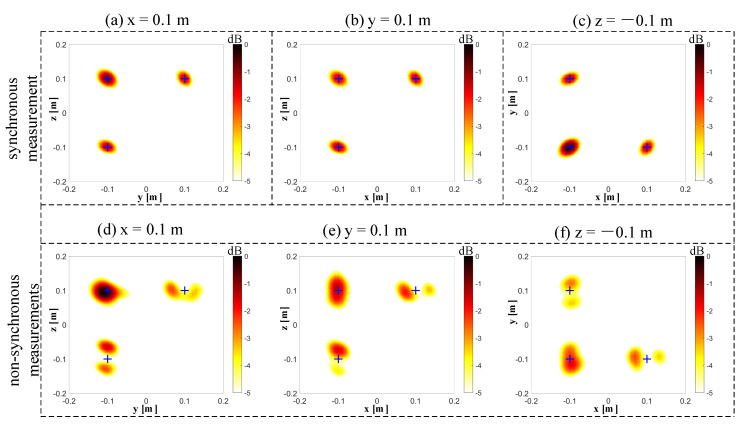
The beamforming results of the synchronous and non-synchronous measurements at 4000 Hz. (**a**) The beamforming map of the synchronous measurement at the x = 0.1 m slice. (**b**) The beamforming map of the synchronous measurement at the y = 0.1 m slice. (**c**) The beamforming map of the synchronous measurement at the z = −0.1 m slice. (**d**) The beamforming map of the non-synchronous measurements at the x = 0.1 m slice. (**e**) The beamforming map of the non-synchronous measurements at the y = 0.1 m slice. (**f**) The beamforming map of the non-synchronous measurements at the z = −0.1 m slice.

**Figure 13:**
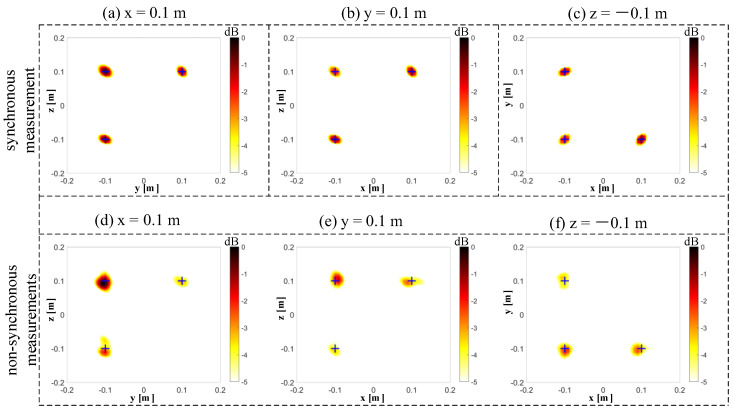
The beamforming results of the synchronous and non-synchronous measurements at 6000 Hz. (**a**) The beamforming map of the synchronous measurement at the x = 0.1 m slice. (**b**) The beamforming map of the synchronous measurement at the y = 0.1 m slice. (**c**) The beamforming map of the synchronous measurement at the z = −0.1 m slice. (**d**) The beamforming map of the non-synchronous measurements at the x = 0.1 m slice. (**e**) The beamforming map of the non-synchronous measurements at the y = 0.1 m slice. (**f**) The beamforming map of the non-synchronous measurements at the z = −0.1 m slice.

**Figure 14:**
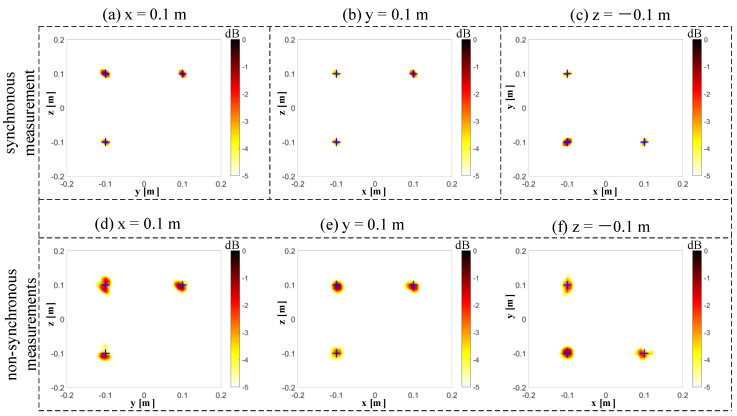
The beamforming results of the synchronous and non-synchronous measurements at 8000 Hz. (**a**) The beamforming map of the synchronous measurement at the x = 0.1 m slice. (**b**) The beamforming map of the synchronous measurement at the y = 0.1 m slice. (**c**) The beamforming map of the synchronous measurement at the z = −0.1 m slice. (**d**) The beamforming map of the non-synchronous measurements at the x = 0.1 m slice. (**e**) The beamforming map of the non-synchronous measurements at the y = 0.1 m slice. (**f**) The beamforming map of the non-synchronous measurements at the z = −0.1 m slice.

The results of 3D imaging between the synchronous and non-synchronous measurements are compared in this section. The microphone array is moved orthogonally twice for the non-synchronous measurements. The corresponding synchronous measurement is that the three microphone arrays capture data simultaneously. Figure 11a shows the cross-spectral matrix from three independent measurements, which has been arranged on the matrix’s diagonal in the form of blocks. The elements in the non-diagonal block positions of the measured spectral matrix are missing, and they have been filled with zeros in this figure to visualize these unknown parts. Figure 11b shows the spectral matrix completion by the proposed non-synchronous measurements method. All elements in the current spectral matrix are known in both diagonal and non-diagonal blocks. Figure 11c shows the full cross-spectral matrix in the case of all microphones in three arrays, capturing data simultaneously (i.e., the synchronous measurements). Let $D^{\mathrm{non}}$ denote the position of the sound source obtained by the non-synchronous measurements. The relative error can quantify the difference between the real sound source position $D^{\mathrm{real}}$ and $D^{\mathrm{non}}$ by the $\ell_{2}\text{-}\mathrm{norm}$, which is obtained as ${\mathrm{Error}}_{\mathrm{position}}=\left({\left\|D^{\mathrm{non}}\right\|}_{2}-{\left\|D^{\mathrm{real}}\right\|}_{2}\right)/{\left\|D^{\mathrm{real}}\right\|}_{2}$. The frequency of beamforming is set to 4000 Hz, and the signal-to-noise ratio is set to 20 dB. In this case, the maximum relative error ${\mathrm{Error}}_{\mathrm{position}}$ of the six source positions is 1.926%. Since the beamforming algorithm is suitable for higher working frequencies, we selected frequencies of 4000 Hz (Figure 12), 6000 Hz (Figure 13), and 8000 Hz (Figure 14). The beamforming map slices of the synchronous measurement are shown in Figure 12a–c, Figure 13a–c, and Figure 14a–c, and the beamforming map slices of the non-synchronous measurements are shown in Figure 12d–f, Figure 13d–f, and Figure 14d–f. The beamforming results of Figure 12, Figure 13 and Figure 14 show (1) both the synchronous measurements and the non-synchronous measurements can accurately locate the position of the sound source and have a good spatial resolution; and (2) compared to the synchronous measurements, the non-synchronous measurements increase the sidelobes slightly, but the number of microphones required for measurement is reduced significantly in the localization of the spatial sound sources.

**Figure 15:**
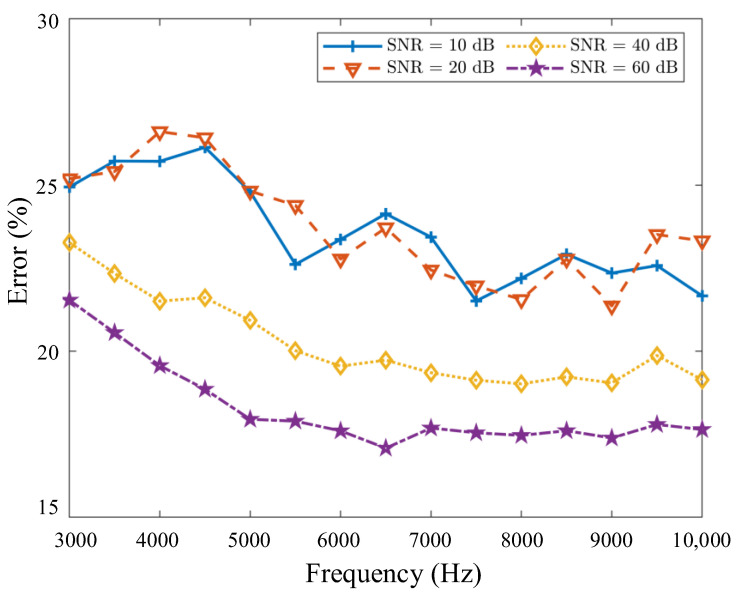
The relative error of the spectral matrix completion ($S^{\mathrm{com}}$) and the full spectral matrix ($S^{\mathrm{Full}}$) with different frequencies and different signal-to-noise ratios (SNRs).

The relative error can quantify the difference between the spectral matrix completion ($S^{\mathrm{com}}$) and the full spectral matrix ($S^{\mathrm{Full}}$) by the Frobenius norm, which is obtained as ${\mathrm{Error}}_{\mathrm{CSM}}=\left({\left\|S^{\mathrm{com}}\right\|}_{F}-{\left\|S^{\mathrm{full}}\right\|}_{F}\right)/{\left\|S^{\mathrm{full}}\right\|}_{F}$. Figure 15 shows the errors of the spectral matrix completion ($S^{\mathrm{com}}$) and the full spectral matrix ($S^{\mathrm{Full}}$) with different frequencies and different SNRs. Although the performance of the non-synchronous measurements is influenced by noise, the errors of $S^{\mathrm{com}}$ and $S^{\mathrm{Full}}$ decrease with the SNR. When the SNR is relatively low (for example, SNR = 20 dB and 10 dB), the noise plays a key role in the results of acoustic reconstruction. As the SNR increases and frequency increase, the difference between the spectral matrix completion and the full spectral matrix gradually decreases.

## 5. Experimental Verification

### 5.1. Experimental Setup

**Figure 16:**
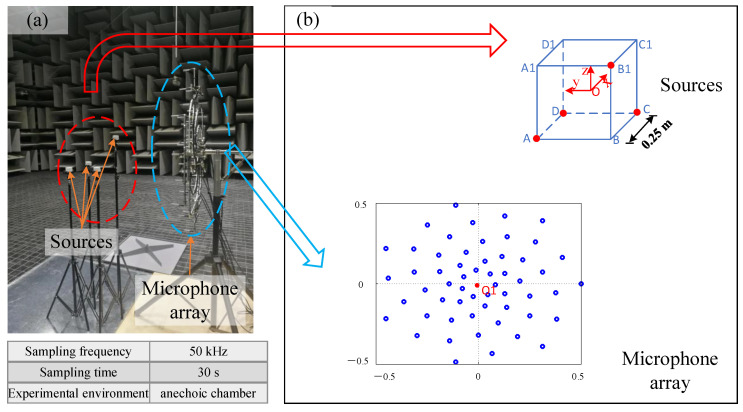
(**a**) The on-site experiment setup. (**b**) The schematic diagram of the sound sources and the microphone array.

**Figure 17:**
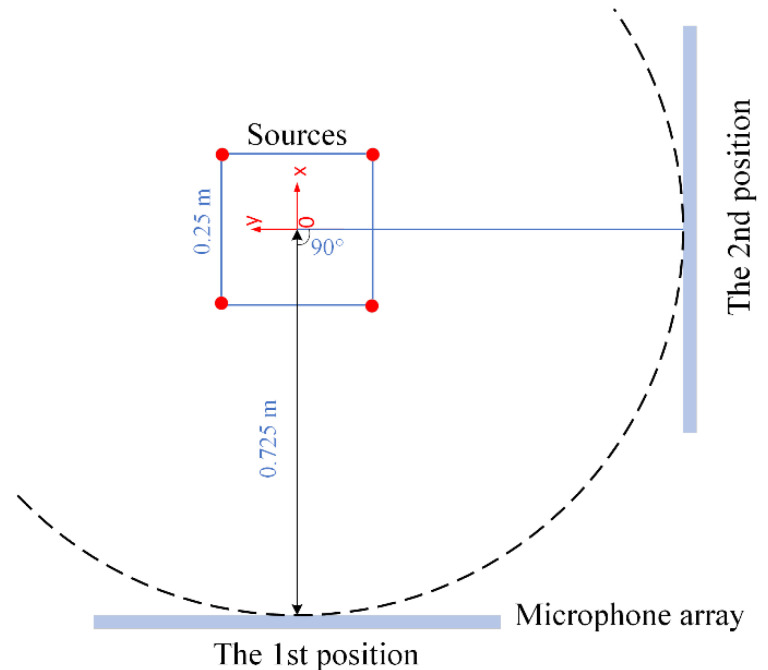
The top view of the moving strategy for the microphone array in the experiment.

An experiment of applying the non-synchronous measurements to 3D acoustic imaging has been done in a full-anechoic chamber with a cut-off frequency of 40 Hz and background noise of −1 dB(A). The acoustic vibration measurement platform was provided by the Zhejiang Institute of Metrology (ZJIM). The microphone model was MPA416 (provided by Beijing Prestige Technology Co., Beijing, China). The acquisition instrument model was DH5922D (provided by Jiangsu Donghua Testing Technology Co., Ltd., Jingjiang, China). Figure 16a shows the photograph of the on-site experiment setup. To display the spatial locations of the sound sources, the schematic diagram of the sound source locations and the geometry of the microphone array are drawn in Figure 16b. Four speakers are not in one plane in the experiment. The sound source was driven by Bluetooth speakers. Similar to previous simulations, these four speakers were located at the four vertices (A, C, D and B1) of an imaginary cube ABCD-A1B1C1D1. The side length of this imaginary cube was about 0.25 m. To more conveniently describe the spatial position of the sources and the array in the following, this imaginary cube’s faces and vertices were used as the reference for positioning. The four speakers were supported by four tripods and placed on the marked locations on the ground. The microphone array has 56 elements and has the same geometry as the previous simulation. Taking the center of the imaginary cube (O) as the origin, the space Cartesian coordinate system is established in Figure 16b. When the microphone array was initially placed, the array was placed parallel to the plane ABB1A1 with a distance of 0.6 m. The microphone array was located on the x = −0.725 m plane. The microphone array position was adjusted so that the position of the array center (O1) is the same as the imaginary cube center (O). The strategy for moving the microphone array is shown in Figure 17. It is the top view. The 1st position of the microphone array was located at the x = −0.725 m plane. In the second measurement, the microphone array was rotated 90 degrees counterclockwise. The 2nd position of the microphone array was located at the y = −0.725 m. During the measurement, four speakers simultaneously emitted 4000 Hz pure tone. The acquisition instrument was provided by Jiangsu Donghua Testing Technology Co., Ltd., and its model was DH5922D. When there were more than 32 channels, the maximum sampling rate was 128 kHz/channel. So, a sampling frequency of 50 KHz was selected. The sampling time was 30 s.

### 5.2. Experimental Results

**Figure 18:**
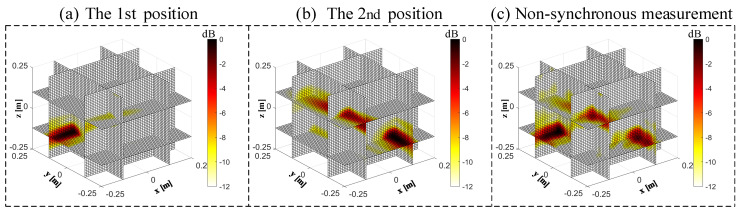
Comparison of the 3D beamforming results between a single planar microphone array and the non-synchronous measurements. (**a**) The 1st position; (**b**) the 2nd position; (**c**) the non-synchronous measurements.

The 3D beamforming results between a single planar microphone array and the non-synchronous measurements are compared in Figure 18. A significant difference is visible in three cases. Only with the planar microphone array in the 1st position (Figure 18a) or 2nd position (Figure 18b), there are one or two distinct maximum peaks in the field of view, and there is a large sidelobe in the normal direction of each position. It is impossible to locate the spatial source sources accurately. With the non-synchronous measurements (Figure 18c), three distinct maximums appear in the field of vision, and the side lobes in the normal direction are reduced significantly. The 4th sound source is located at the corner on the other side, which was blocked by the slices of the 3D beamforming. The spatial localization accuracy of the sound sources is significantly improved when using the non-synchronous measurements when moving the arrays in 3D space, especially when the microphone array is perpendicular to the sound source plane.

**Figure 19:**
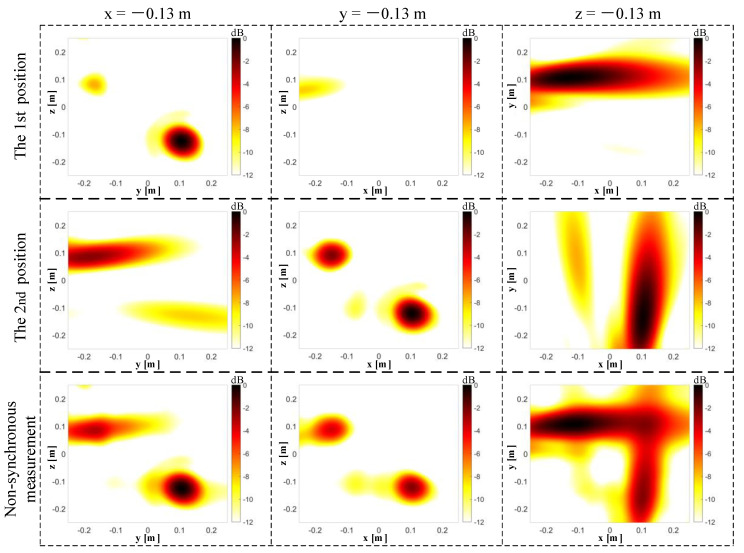
Comparison of the beamforming slices between a single planar microphone array and the non-synchronous measurements. From top to bottom are the 1st position, the 2nd position, and the non-synchronous measurements. From left to right are the x = −0.13 m, y = −0.13 m, and z = −0.13 m slices.

Three slices (x = −0.13 m, y = −0.13 m, z = −0.13 m) of the 3D beamforming results are plotted in Figure 19. With the single microphone array placed in the 1st position (i.e., parallel to the y-z plane), two sound sources can be located at the upper-left and lower-right quadrants at the x = −0.13 m plane. At the y = −0.13 m and z = −0.13 m plane, beamforming maps are not used to determine the sources’ positions due to the larger extension of the lobes. With the single microphone array placed in the 2nd position (i.e., parallel to the x-z plane), the beamforming map at the y = −0.13 m plane exhibits two major lobes that correspond to the positions of the two sound sources. At the x = −0.13 m and z = −0.13 m plane, the main lobes extend so badly that it is impossible to find the sound source positions exactly. When the non-synchronous measurements are adopted, the spatial resolution in both the x = −0.13 m and y = −0.13 m planes is improved relative to the single microphone array. For the z = −0.13 m plane, the beamforming map is improved slightly with the non-synchronous measurements than with the single microphone array. Comparing the beamforming between the non-synchronous measurements and the single microphone array suggests that the non-synchronous measurements improve the spatial positioning resolution of the planar microphone array.

## 6. Conclusions

Due to the scanning area being a plane in the 2D beamforming method, the distance between the microphone and the sound source needs to be known in advance. The sound sources of industrial production are randomly distributed in space, instead of in a plane. The application of conventional beamforming from 2D to 3D is expanded in this paper. By moving the microphone array in space, the non-synchronous measurements can significantly improve the planar array’s positioning resolution in the normal direction. The 3D sound source localization is achieved by increasing the number of microphone arrays in most current studies, which have the disadvantage of requiring more microphones and acquisition channels. The non-synchronous measurements overcome this shortcoming and can also achieve a high-resolution in 3D positioning. The method proposed in this paper has extensive application potential in the field of 3D sound localization.
